# Optimization of Density Peak Clustering Algorithm Based on Improved Black Widow Algorithm

**DOI:** 10.3390/biomimetics9010003

**Published:** 2023-12-21

**Authors:** Huajuan Huang, Hao Wu, Xiuxi Wei, Yongquan Zhou

**Affiliations:** 1 College of Artificial Intelligence, Guangxi Minzu University, Nanning 530006, China; hhj@gxmzu.edu.cn (H.H.); liaoheng@stu.gxmzu.edu.cn (H.W.); yongquanzhou@126.com (Y.Z.); 2 School of Computer Science & Technology, China University of Mining and Technology, Xuzhou 221116, China; 3 Guangxi Key Laboratory of Hybrid Computation and IC Design Analysis, Guangxi Minzu University, Nanning 530006, China

**Keywords:** clustering, density peak clustering, cutoff distance, black widow algorithm

## Abstract

Clustering is an unsupervised learning method. Density Peak Clustering (DPC), a density-based algorithm, intuitively determines the number of clusters and identifies clusters of arbitrary shapes. However, it cannot function effectively without the correct parameter, referred to as the cutoff distance (*d_c_*). The traditional DPC algorithm exhibits noticeable shortcomings in the initial setting of *d_c_* when confronted with different datasets, necessitating manual readjustment. To solve this defect, we propose a new algorithm where we integrate DPC with the Black Widow Optimization Algorithm (BWOA), named Black Widow Density Peaks Clustering (BWDPC), to automatically optimize *d_c_* for maximizing accuracy, achieving automatic determination of *d_c_*. In the experiment, BWDPC is used to compare with three other algorithms on six synthetic data and six University of California Irvine (UCI) datasets. The results demonstrate that the proposed BWDPC algorithm more accurately identifies density peak points (cluster centers). Moreover, BWDPC achieves superior clustering results. Therefore, BWDPC represents an effective improvement over DPC.

## 1. Introduction

Clustering is a type of unsupervised learning method [1] that plays a crucial role in extracting essential and potentially valuable information from data [2]. It is a typical unsupervised learning technique that aggregates data objects into clusters based on some similarity measure without the need for any prior knowledge. Without the need for any prior knowledge about the data, data objects within the same cluster demonstrate high similarity, while those in different clusters exhibit low similarity in the dataset. Leveraging these advantages, clustering has been applied in various fields, such as community detection [3], pattern recognition [4], image processing [5], financial services [6], and security detection [7], and has achieved success in these domains.

With the rapid advancement of data science and machine learning, cluster analysis has evolved into a fundamental technique in the realms of data mining and pattern recognition. The development of clustering algorithms has progressed through several crucial stages. Initially, clustering algorithms primarily revolved around distance-based hierarchical methods like Clustering using representative (CURE) [8], Balanced iterative reducing and clustering using hierarchies (BIRCH) [9], and partition-based approaches such as K-means [10] and K-medoids [11] and Gaussian Mixed Model (GMM) [12]. Despite their effectiveness on simple datasets, these algorithms faced limitations in handling large-scale and high-dimensional data. As data sizes and complexity increased, researchers started developing more sophisticated clustering algorithms. A notable advancement came with the introduction of density-based clustering algorithms, exemplified by Density-Based Spatial Clustering of Applications with Noise (DBSCAN) [13] and Or-dering Points to Identify the Clustering Structure (OPTICS) [14]. These algorithms eschew reliance on global distance measures; instead, they ascertain cluster structures based on the density of data points. Compared with traditional clustering algorithms, density-based clustering algorithms have greater advantages in finding clusters of arbitrary shapes and sizes and in pro-cessing data of complex shapes [15,16]. Nonetheless, Density-based clustering algorithms have their drawbacks [17,18], including sensitivity to parameters, issues with density differences, and challenges in managing high-dimensional data environments.

Density Peak Clustering (DPC) [19] is a density-based clustering algorithm proposed by Alex Rodriguez and Alessandro Laio in 2014. This algorithm identifies density peaks in a dataset by calculating the local density and distance of data points and assigns them to different clusters. DPC is a valuable tool in data analysis, revealing the underlying structure and patterns that facilitate a deeper understanding of the data. In computer vision, DPC finds applications in image segmentation, aiding in the identification of distinct objects or areas within an image. DPC can quickly discover density peak points (cluster centers) of datasets with arbitrary shapes, efficiently allocating sample points to clusters and removing outliers. However, DPC has limitations in capturing complex-shaped multimodal clusters [20,21] because the assumption about cluster centers does not provide a reliable criterion for identifying true density peaks. As a result, incorrect center selection leads to poor clustering results. Furthermore, DPC exhibits high sensitivity to parameters, with the clustering outcomes frequently affected by the value of *d_c_*. Varied *d_c_* values may result in distinct clustering outcomes. Additionally, the DPC algorithm tends to underperform on high-dimensional data, failing to produce satisfactory clustering results. To address this issue, researchers have conducted studies to enhance DPC. Chen [22] proposed a DPC algorithm based on Chebyshev inequality and differential privacy (DP-CDPC); the DP-CDPC algorithm can effectively select the clustering centers, improve the quality of clustering results, and provide good privacy protection performance. Wu [23] proposed a quantum DPC (QDPC) algorithm based on a quantum DistCalc circuit and a Grover circuit. It can effectively improve the efficiency of dealing with a large number of data scenarios, and it is easier to pinpoint the parameter range. In order to further improve the operation efficiency of DPC, Jiang [24] combined the artificial bee colony algorithm with density peak clustering to propose an enhanced clustering algorithm. This algorithm effectively utilized the global and local search capabilities of the artificial bee colony algorithm, resulting in higher quality and more stable clustering results. Li [25] integrated the particle swarm optimization algorithm with density peak clustering, presenting a dynamic optimization algorithm based on automatic and fast density peak clustering. It created parameter-insensitive subpopulations based on particle self-density and relative distance, improving the accuracy and robustness of clustering results.

The Black Widow Algorithm [26] is an optimization algorithm based on principles from biology, inspired by the mating behavior of black widow spiders. This algorithm simulates different movement strategies employed by black widow spiders during the mating process to search for optimal solutions. By introducing various motion strategies such as crawling, crouching, and swinging, the algorithm imitates the spider’s different behaviors in the mating process. These motion strategies enable the spider to explore the search space locally and globally, gradually optimizing the individual’s position to find the best solution. Moreover, the Black Widow Algorithm considers the role of pheromones; spiders release and perceive pheromones to influence the movement strategies and behaviors of other spiders, thus cooperating and collaborating within the group to achieve better search results. The Black Widow Algorithm exhibits good search performance and convergence and is suitable for various optimization problems, including function optimization, parameter optimization, and machine learning tasks. By simulating the mating behavior and pheromone communication of black widow spiders, this algorithm efficiently finds optimal solutions and demonstrates promising performance in practical applications.

The reference [19] does not provide a method for calculating the cutoff distance “*d_c_*” but leaves it to the users to define manually. To overcome the problem of manual selection and achieve automatic optimization of the DPC algorithm’s cutoff distance “*d_c_*”, this paper proposes a Density Peak Clustering algorithm based on the Black Widow Optimization Algorithm (BWDPC). The BWDPC clustering algorithm aims to select the optimal “*d_c_*” value using the Silhouette Coefficient (Sil) as the optimization objective. The algorithm follows a selection process where, within a certain number of iterations, it chooses the “*d_c_*” value corresponding to the highest Sil. This process helps to identify the best density centers under a reasonable “*d_c_*” setting. The results obtained from synthetic datasets and UCI real datasets demonstrate that the BWDPC algorithm can correctly select density centers. The main contributions of the BWDPC algorithm are as follows:

1. Intelligent Optimization with Sil Objective: By using an intelligent optimization algorithm with the Silhouette Coefficient as the objective, the BWDPC algorithm overcomes the problem of inaccurate density center selection in previous DPC algorithms, which could lead to chain errors in the clustering results.
2. Improved Black Widow Algorithm: The traditional Black Widow Algorithm has been modified by incorporating search factors, making it more suitable for optimizing the DPC algorithm. Multiple rounds of swarm intelligence search have been conducted to address issues such as the algorithm’s limited search paths and slow convergence.
3. Automatic Selection of “*d_c_*”: BWDPC requires only the initialization of “*d_c_*”, and then it automatically selects the appropriate “*d_c_*” value during the clustering process. This feature makes it well-suited for handling large-scale datasets.

The main content of this paper is as follows: Section 2 introduces related works. Section 3 primarily discusses the proposed BWDPC method. Section 4 presents experiments and discussions. Section 5 presents the conclusions.

## 2. Related Works

### 2.1. The DPC Algorithm

The DPC algorithm is based on two intuitive assumptions:

1. Points around a clustering center have lower densities than the center itself.
2. Clustering centers are farther away from other points with higher densities. The algorithm requires the input of a cutoff distance parameter *d_c_*. It then automatically selects clustering center points on the decision graph based on the given *d_c_* value. Afterward, using a one-step allocation strategy, it assigns the remaining points to the clusters represented by the identified centers to complete the clustering process.

#### 2.1.1. The Relevant Parameters of the DPC Algorithm

In the DPC algorithm, there are two different ways to calculate the local density based on the size of the dataset. When the dataset is large, the calculation of local density is as follows:

(1) $$\rho_{i}=\sum_{i\neq j}\chi \left(d_{ij}-d_{c}\right)$$

(2) $$\rho_{i}=\{\begin{array}{c} \chi \left(x\right)=1\;,\;x\;<\;0\\ \chi \left(x\right)=0\;,\;x\geq 0 \end{array}$$

where *d_c_* is a cutoff distance and $\rho_{i}$ represents the local density of point *i*. $d_{ij}$ denotes Euclidean distance between point *i* and point *j*, and *x* is equivalent to $d_{ij}$ minus *d_c_*. χ(x)=1 when *x* < 0, χ(x)=0 when x≥0 . $d_{ij}$ is defined as follows:

(3) $$d_{ij}=\sqrt{\sum_{k=1}^{n}{\left(x_{ik}-x_{jk}\right)}^{2}}$$

where *i* represents point *i*, *j* represents point *j*, and *k* represents the dimension of a certain point. When the dataset size is small, the definition of local density is as follows:

(4) $$\rho_{i}=\sum_{j\neq i}\exp\left(-\frac{{d_{ij}}^{2}}{{d_{c}}^{2}}\right)$$

where the cutoff distance is the only parameter of DPC. In DPC, *d_c_* = M_sort_(round(*pn*)). M_sort_ represents an ordered set of all values in the distance matrix M from small to large. The value of *p* is about 1% to 2%.

Set δ as the center deviation distance and δ as the center deviation distance of each point. The center deviation distance represents the minimum distance between point *x_i_* and the set of points whose local density is locally larger than *x_i_*. For point *x_i_* with the largest local density value, $\delta_{i}$ represents the maximum distance between that point and other points. The definition of center deviation distance can be expressed as in mathematical Equation (5).

(5) $$\delta_{i}=\left\{ \begin{array}{l} \underset{j}{\min}(d_{ij}),\rho_{j}>\rho_{i}\\ \underset{j}{\max}(\delta_{i}),\mathrm{otherwise} \end{array}\right.$$

Given a dataset *X* = {*x_1_*, *x_2_*, …, *x_n_*} for ∀x_i_ ∈ *X*, the decision value for *x_i_* is calculated as follows:

(6) $$\gamma_{i}=\rho_{i}\;\cdot \;\delta_{i}$$

where $\rho_{i}$ is the local density of *x_i_*,*δ_i_* is the relative distance of *x_i_*, and $\gamma_{i}$ is the decision value of point *i*.

**Figure 1:**
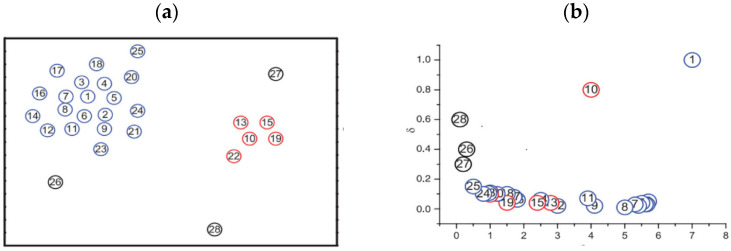
(**a**) Data distribution diagram; (**b**) decision graph based on ρ and *δ*.

As shown in Figure 1, the blue point is a class cluster, and the red point is another class cluster. After calculating the ρ and δ values based on the aforementioned steps for each point, points 1 and 10 have the highest γ values, and it can be seen that sample points 1 and 10 are located at the upper right corner of the decision diagram. Therefore, points 1 and 10 are identified as cluster centers. The black points 26, 27, and 28 have a relatively high δ and a low ρ because they are isolated, and they can be considered outliers. Clustering centers should have large δ and ρ .

The clustering process of DPC is as follows:

1. Calculate the local density ( ρ ) and relative distance (*δ*) of each sample point using Equations (1)–(5).
2. Calculate the decision value for each sample point using Equation (6).
3. Select the points with higher decision values as the cluster centers.
4. Once the cluster centers are identified, allocate the remaining points in descending order of their local density. Each point is sequentially assigned to the cluster of the nearest preceding point in terms of relative distance.

DPC shows greater sensitivity to the initial selection of cluster centers compared to the K-means. Different initial values can yield diverse clustering results. In contrast to K-means, DPC eliminates the need for initializing cluster centers, rendering its clustering outcomes insensitive to initial conditions. DBSCAN may have poorer clustering results in situations with significant density variations, while DPC excels in handling clusters with different densities because it determines clusters through local density peaks.

#### 2.1.2. The Limitations of DPC

**Figure 2:**
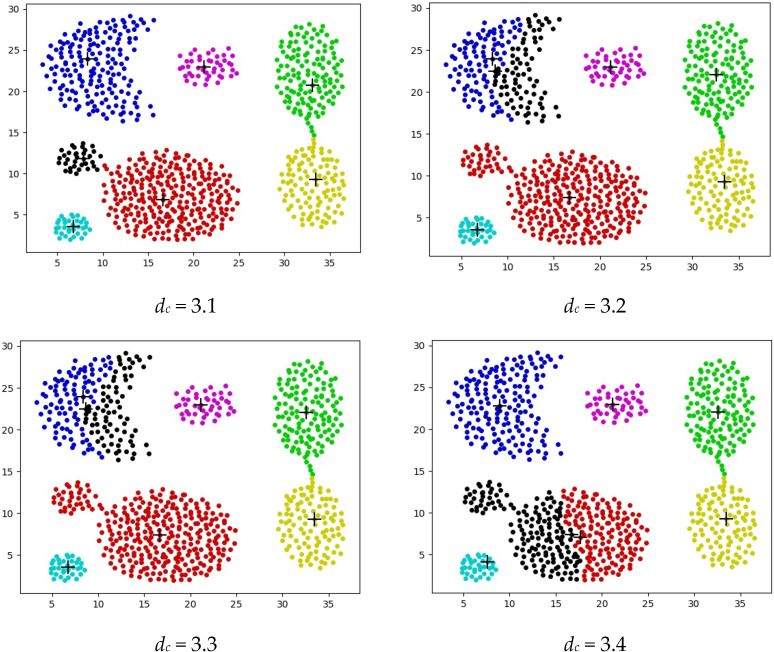
Clustering result graphs corresponding to different values of *d_c_*.

**Figure 3:**
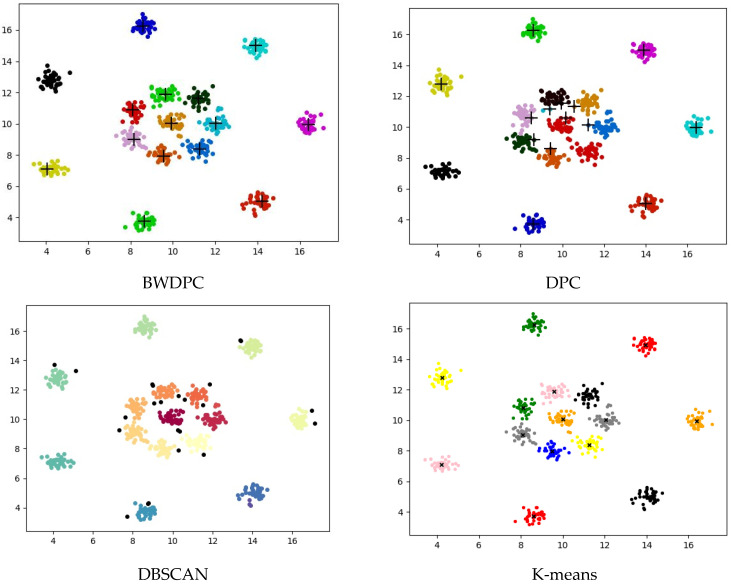
The clustering results of the four algorithms on the R15 dataset.

**Figure 4:**
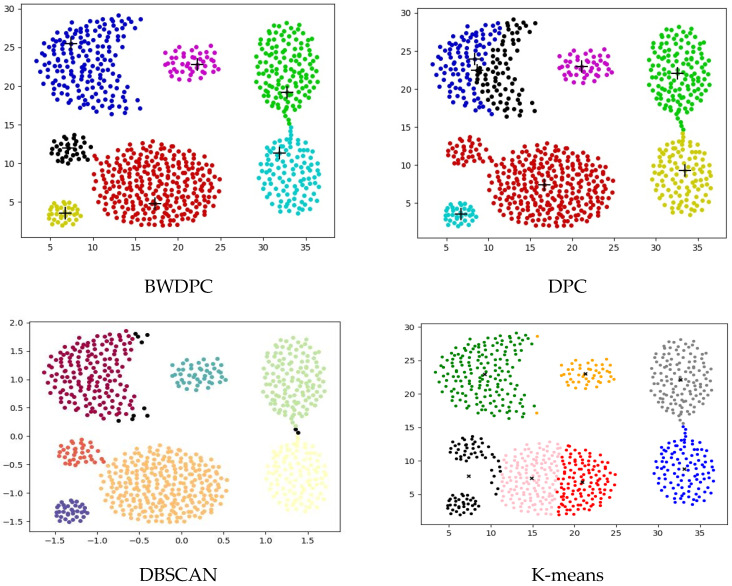
The clustering results of the four algorithms on the Aggregation dataset.

**Figure 5:**
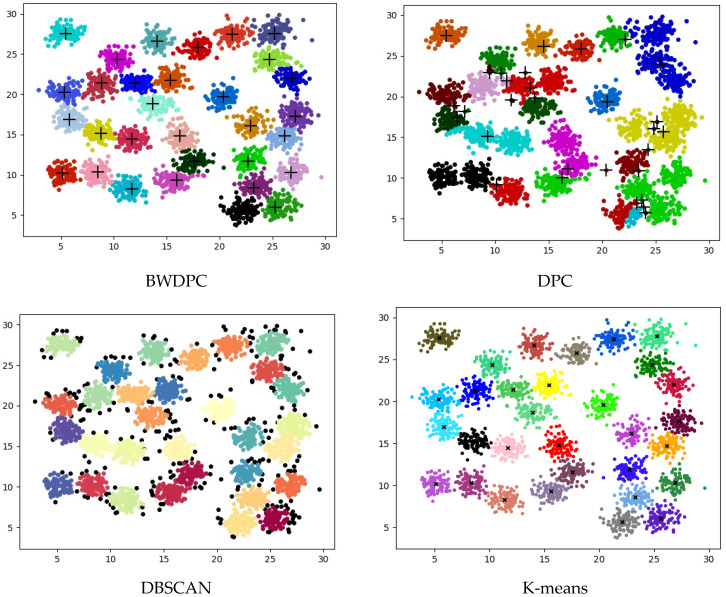
The clustering results of the four algorithms on the D31 dataset.

**Figure 6:**
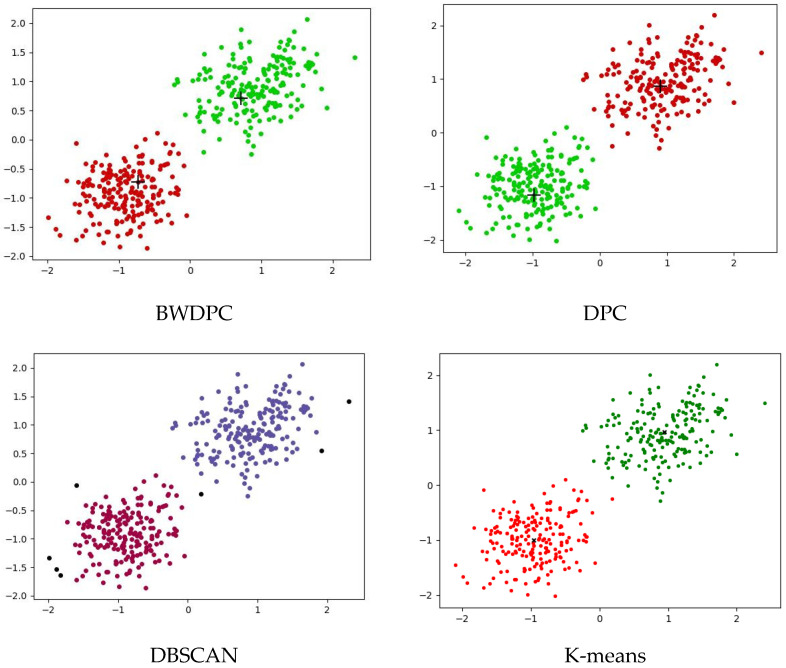
The clustering results of the four algorithms on the Two_cluster dataset.

**Figure 7:**
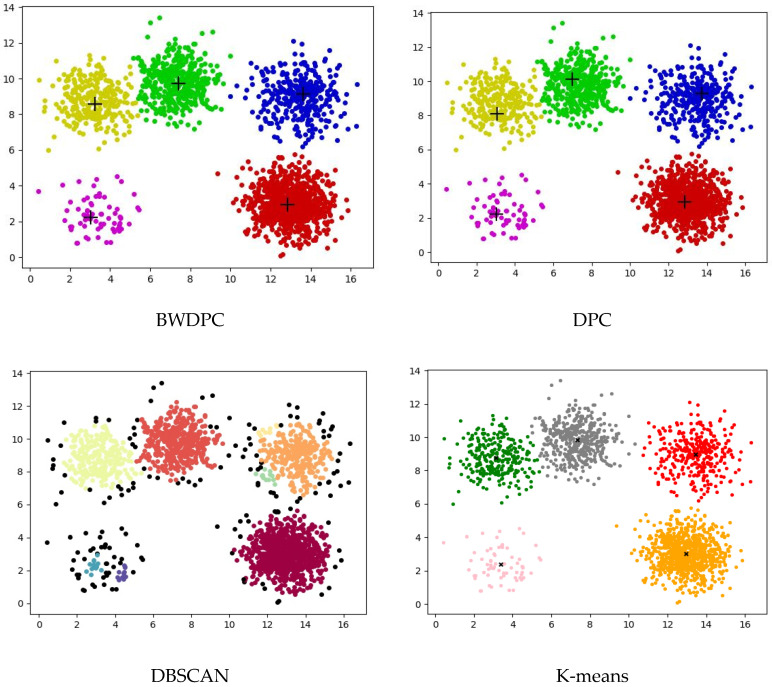
The clustering results of the four algorithms on the Five_cluster dataset.

The idea of the DPC algorithm is relatively simple, as it can recognize clusters of arbitrary shapes and intuitively determine the number of clusters. However, it still has the following shortcomings:

The cutoff distance *d_c_* value needs to be manually set, and its selection is quite sensitive. To illustrate this issue more intuitively, take the clustering results obtained from the Aggregation dataset shown in Figure 2 as an example. It can be observed that different values of *d_c_* lead to significantly different clustering results. Therefore, optimizing the cutoff distance *d_c_* becomes particularly important. The "+" in Figure 2, Figure 3, Figure 4, Figure 5, Figure 6 and Figure 7 represents the cluster center, and samples of different types of clusters are represented by different colors.

As shown in Figure 2, when the cutoff distance (*d*_c_) is set to 3.1, the DPC correctly divides the Aggregation dataset into seven clusters, yielding satisfactory clustering results. However, with *d_c_* set to 3.2, 3.3, and 3.4, the clustering effect diminishes. For instance, with *d_c_* = 3.2, points from the same cluster in the upper-left corner are incorrectly assigned to two clusters, and those from two clusters in the lower-left corner are assigned to the same cluster, resulting in suboptimal clustering and performance. Similar issues arise at *d_c_* = 3.3, where points belonging to the red cluster are incorrectly assigned to the black cluster. Hence, optimizing the truncation distance *d_c_* is crucial for DPC, and the optimal value varies across datasets, influencing clustering outcomes.

### 2.2. BWOA Algorithm

#### 2.2.1. Spider Movement

The movement of spiders in the spiderweb is modeled in two forms: linear and spiral. The position update formula is as follows:

(7) $$\vec{x_{i}}\left(t+1\right)={\vec{x}}_{*}-m{\vec{x}}_{r1}\left(t\right)\;\;\;\;\;\;\;\;\;\;\;\;\mathrm{if}\;\mathrm{rand}\;<\;0.3$$

(8) $$\vec{x_{i}}\left(t+1\right)={\vec{x}}_{*}-\cos\left(2\pi \beta \right)m{\vec{x}}_{i}\left(t\right)\;\;\;\;\;\;\mathrm{otherwise}$$

where ${\vec{x}}_{*}\;$ is the position of the current best individual, *m* is a random floating-point number between 0.4 and 0.9, and *β* is a random floating-point number between −1 and 1. *m* and β are random parameters, and their purpose is to ensure the randomness of the black widow’s movement and to prevent falling into local optimality. *t* represents the generation of the black widow spider, ${\vec{x}}_{r1}\left(t\right)$ is the position of the *r*1 black widow, and ${\vec{x}}_{i}\left(t\right)$ is the current position of the black widow. The rand value falls within the range of (0, 1), ensuring the randomness of the black widow spider’s movement. Assuming the spider moves in a linear and spiral fashion within the grid, it follows Formula (7) when rand <0.3; otherwise, it adheres to Formula (8).

#### 2.2.2. Pheromone

Pheromones play a crucial role in the mating process of spiders. The correlation between spider diet and the variation in pheromone signals affecting the quality and quantity of silk is evident. Male spiders are more sensitive to the sex pheromones secreted by well-nourished females because they indicate higher reproductive capability, but mainly to avoid the cost of mating with potentially starved females. Therefore, male spiders tend to avoid females with low pheromone content. The pheromone deposition rate for black widow spiders can be calculated according to Formula (9).

(9) $$pheromone(i)=\frac{fitness_{\max}-fitness\left(i\right)}{fitness_{\max}-fitness_{\min}}$$

When the pheromone value is less than or equal to 0.3, the individual will be replaced, and the position update formula is as follows:

(10) $${\vec{x}}_{i}(t)=\vec{x}\times \left(t\right)+\frac{1}{2}\left[{\vec{x}}_{r1}\left(t\right)-{\left(-1\right)}^{\sigma }\times {\vec{x}}_{r2}\left(t\right)\right]$$

where *x_r_*_1_ and *x_r_*_2_ are two different individuals, and σ is either 0 or 1. ${\vec{x}}_{i}(t)$ indicates the location of a black widow with low pheromone levels in the female. Additionally, σ is a random number in order to randomize the location of black widows with low pheromone levels in females. ${\vec{x}}_{r1}\left(t\right)$ is the position of the r_1_ black widow, ${\vec{x}}_{r2}\left(t\right)$ is the position of the *r*2 black widow. It is specified that *r*1 must not be equal to *r*2.

### 2.3. Abbreviations and Their Descriptions

In this paper, external clustering evaluation is employed as the objective function, along with several commonly used clustering evaluation metrics, to assess the performance of clustering algorithms. The indices used include the Silhouette Coefficient [27], the Fowlkes–Mallows Index (*FMI*) [28], the Adjusted Rand Index (*ARI*) [29], and Adjusted Mutual Information (*AMI*) [30]. The silhouette refers to a method that reflects the consistency of data clustering results and can be used to evaluate the dispersion between clusters after clustering; others are employed to evaluate the performance of clustering algorithms, offering quantitative measures of the agreement between clustering results and true class labels. The Fowlkes–Mallows Index (*FMI*) exhibits sensitivity to outliers, while the Adjusted Rand Index (*ARI*) demonstrates robustness in handling random clustering results, rendering it more practical in stochastic scenarios. The Adjusted Mutual Information (*AMI*) remains insensitive to dataset size, making it suitable for datasets of various scales. The four evaluation indices are described in detail as follows:

(1) Silhouette Coefficient (*Sil*):

The Silhouette Coefficient is a method to examine how similar an object is to its own cluster compared to other clusters. A data ser D with *n* sample points was divided into *k* clusters: C = (C_1_, C_2_, …, C_K_, …, C_N_). *a*(*t*) could be the average dissimilarity of sample *t* in C_j_. D(*t*, C_i_) was the average dissimilarity of sample point t to all samples in another cluster Ci, then *b*(*t*) = min{d(*t*, C_i_)}, where *i* ≠ *j*. The calculation formula of sample *t* Silhouette index *Sil* was shown in Formula (11).

(11) $$Sil(t)=\frac{\left[b(t)-a(t)\right]}{\max\{a(t),b(t)\}}$$

*Sil*(*t*) value reflected among cluster Ci with compact classes and separable classes. The average of all the samples *Sil*(*t*) values reflect the quality of clustering results. The greater the average *Sil* value, the more compact the class and the better the quality of clustering is. *Sil* has a value range of [−1, 1].

(12) $$Sil=\frac{1}{N}\sum_{t=1}^{N}Sil(t)$$

*Sil* represents the average silhouette value of all sample points, and *N* represents the total number of samples.

(2) Fowlkes–Mallows Index (*FMI*):

The Fowlkes–Mallows Index is defined as follows:

(13) $$FMI=\frac{TP}{\sqrt{\left(TP+FP)(TP+FN\right)}}$$

*TP* represents the count of sample pairs correctly assigned to the same cluster. *FP* represents the count of sample pairs that, according to the true labels, do not belong to the same category but are incorrectly assigned to the same cluster. *FN* represents the count of sample pairs that, according to the true labels, belong to the same category but are incorrectly not assigned to the same cluster. The value of *FMI* ranges from 0 to 1, with a higher value indicating a better clustering result.

(3) The Adjusted Rand Index (*RI*):

The Rand Index (*RI*) is defined as follows:

(14) $$RI=\frac{TP+TN}{TP+FP+TN+FN}$$

TN represents the number of sample pairs that do not belong to the same category in the true labels and are correctly not assigned to the same cluster. The Adjusted Rand Index (*ARI*) is adjusted, and its definition is as follows:

(15) $$ARI=\frac{RI-E(RI)}{\max(RI)-E(RI)}$$

where *E*(⋅) represents the expected value, and the *ARI* has a range of [−1, 1]. The closer the value is to 1, the higher the clustering accuracy is indicated.

(4) Adjusted Mutual Information (*AMI*):

Similar to *ARI*, *AMI* is another widely used cluster evaluation indicator, and its definition is as follows:

(16) $$AMI=\frac{MI-E(MI)}{\max(H(A),H(B)-E(MI))}$$

where *H*(*A*) and *H*(*B*) denote the entropy of two category labels, and *AMI* assesses the clustering effect based on mutual information (*MI*). *E*(*MI*) represents the mathematical expectation of *MI*. *MI*, as a measure of the coincidence of two data distribution indices, is expressed in its tabulated formula:

(17) $$MI(y_{true},y_{pred})=\sum_{i=1}^{k}\sum_{i=1}^{k}p_{ij}\log(\frac{p_{ij}}{p_{i}.p_{j}})$$

*p_ij_* = *m_ij_*/*N*, where *k* is the total number of clusters, *m_ij_* is the number of samples in the intersection of the sample sets for cluster *i* in the true labels and cluster *j* in the clustering algorithm, and *N* is the total number of samples. *p_i_* represents the ratio of the number of samples in cluster *i* in the true labels to the total number of samples *N*, and *p_j_* represents the ratio of the number of samples in cluster *j* in the clustering algorithm to the total number of samples, *N*. The *AMI* has the same range as the *ARI*, and a higher value indicates more accurate clustering results.

The DPC algorithm, based on density peaks, operates without the need for a predefined number of clusters. It dynamically determines the number of clusters by analyzing density relationships between data points, showcasing adaptability to clusters of diverse shapes and sizes. Furthermore, as DPC identifies clusters through density peaks, it demonstrates strong adaptability to clusters with irregular shapes. Compared to certain agglomerative methods, DPC excels in capturing clusters with varying density distributions. Nevertheless, as illustrated in Figure 2, the DPC algorithm exhibits high sensitivity to the *d_c_* parameter. In the subsequent work, the project team successfully conducted experiments by optimizing this parameter. In the subsequent work, the project team optimized this parameter using the Black Widow Algorithm and conducted relevant experiments.

## 3. The Clustering of BWDPC

For the traditional DPC algorithm, the selection of the cutoff distance *d_c_* heavily relies on manual configuration and lacks intelligent optimization. Therefore, this paper proposes the BWDPC algorithm, which can automatically acquire more optimal values, thereby achieving accurate classification. In this section, the improvements of the BWOA algorithm and its combination with the DPC algorithm are elaborately presented.

### 3.1. The Shortcomings of the BWOA

The Black Widow Algorithm is an innovative population optimization algorithm inspired by the unique mating behavior of black widow spiders. It features minimal control parameters, straightforward operation, and strong optimization performance. Despite showing good performance in certain cases, this algorithm also has some drawbacks and limitations, including difficulties in parameter selection, susceptibility to local optima, problem-specific nature, and slow convergence speed, among others.

### 3.2. The RBWOA Model

The conventional BWOA algorithm exhibits slow convergence, particularly in addressing complex and high-dimensional optimization problems. It is susceptible to entrapment in local optima, with a weak search capability. To tackle these challenges, and informed by the issues identified in the proposed DPC algorithm, we devise a strategy to dynamically update the spider population range, capitalizing on the characteristics of the sigmoid function [30].

(18) $$u=\frac{1}{1+e^{-t}}$$

(19) $$S_{i+1}=S_{i}-\;r\;\times \;u\;\times {\;S}_{i}$$

where u represents the search factor, *t* is the number of iterations, *S* represents the spider population range, and *r* is a random number between 0 and 1. *S_i_* denotes the maximum number of generation *i* black widow spiders. In this equation, it can be observed that the spider population range decreases with an increase in the number of iterations, and *r* is introduced to enhance the randomness of the search. In the initial iterations, with small values of *t* and the objective function u, the spider population range is relatively large. As the number of iterations grows and the objective function u increases, the spider population range nonlinearly decreases, gradually approaching 0. This effectively speeds up convergence while preventing entrapment in local optima.

### 3.3. Regarding the Pseudocode for BWDPC

The RBWOA algorithm (Algorithm 1) is as follows.
**Algorithm 1:** RBWOA algorithm
$\mathrm{Input}:\;\mathrm{The}\;\mathrm{value}\;\mathrm{of}\;\mathrm{the}\;\mathrm{initial}\;\mathrm{population}\;S\;\mathrm{Output}:\;\mathrm{The}\;\mathrm{optimal}\;\mathrm{value}\;\mathrm{of}\;x.\;1.\;\mathrm{Initialize}\;\mathrm{the}\;\mathrm{population}\;\mathrm{and}\;\mathrm{evaluate}\;\mathrm{the}\;\mathrm{fitness}\;\mathrm{function}\;\mathrm{values}\;\mathrm{and}\;\mathrm{population}\;S\;\mathrm{based}\;\mathrm{on}\;\mathrm{Formula}\;(9).\;2.\;\mathrm{Generate}\;\mathrm{random}\;\mathrm{parameters}\;m,\;\beta \;3.\;r1=\mathrm{random}.\mathrm{uniform}(0,S),\;r2=\mathrm{random}.\mathrm{uniform}(0,S),\;{{\mathrm{fit}}_{\max}}_{\;}=1,\;{\mathrm{fit}}_{\min}=-1\;4.\;\mathrm{if}\;\mathrm{rand}\;\leq 0.3$

$5.\;\;\vec{x_{i}}\left(t+1\right)={\vec{x}}_{*}-m{\vec{x}}_{r1}\left(t\right)$

$6.\;\mathrm{else}:\;7.\;\;\vec{x_{i}}\left(t+1\right)={\vec{x}}_{*}-\cos\left(2\pi \beta \right)m{\vec{x}}_{i}\left(t\right)$
8. *fit* = *Sil* // Calculate the fitness value “*fit*” using *Sil*
9. *pheromone* = (*fit_max_* − *fit*) / (*fit_max_* − *fit_min_*) // Calculate the fitness value “*fit*” using *Sil* 
10. if pheromone ≤0.3:

$11.\;\;\;\;{\vec{x}}_{i}(t)=\vec{x}\times \left(t\right)+\frac{1}{2}\left[{\vec{x}}_{r1}\left(t\right)-{\left(-1\right)}^{\sigma }\times {\vec{x}}_{r2}\left(t\right)\right]$

$12.\;\mathrm{if}\;{\vec{x}}_{i}\left(t\right)$

≤0:

$13.\;//\mathrm{When}\;{\vec{x}}_{i}\left(t\right)$
 is less than or equal to 0, it indicates the current population *S_i_* has been searched, and iterate to the next population *S_i + 1_*. 14.  *u* = 
$\frac{1}{1+{\mathrm{e}}^{-t}}$
15.  
$S_{i+1}=S_{i}-r\times u\times S_{i}$

$16.\;\mathrm{return}\;\mathrm{to}\;\mathrm{step}\;2\;17.\;\mathrm{else}:\;18.\;\mathrm{output}\;{\vec{x}}_{i}\left(t+1\right)$



### 3.4. Algorithm Flow Steps

The specific flow of the BWDPC algorithm is presented in the table below, incorporating search factors and path optimization search strategies to enhance the algorithm’s convergence speed and efficiency. The BWDPC algorithm (Algorithm 2) is as follows.
**Algorithm 2:** BWDPC algorithmInput: Experimental Dataset *X* = {*x_1_, x_2_, …, x_n_*} Output: Clustering Results *C* = {*c_1_, c_2_, …, c_m_*}, *m* Is the Number of Data Cluster Results
1. Set the population size *S* and the maximum number of iterations *T* for the BWDPC algorithm
2. Data preprocessing: Calculate the distance matrix for all data points and determine the range of *d_c_* values
3. Enter *S* into BWDPC and set the output x of BWDPC to *d_c_*
4. Substitute *d_c_* into equations 4 and 5 to calculate the local density 
ρ
_i_ and 
$\delta_{i};$
 for all points
5. Formula (6) is employed to calculate *γi*, and the initial *m* points with the highest *γ_i_* values are automatically chosen as the cluster centers
6. Introduce the evaluation metric Sil as the objective function for BWDPC and record the *d_c_* value d* corresponding to the maximum *Sil*
7. Check if the termination condition is met. If *t* > *T*, end the iteration and proceed to step
8. If not, go back to step 3 for further optimization
9. Output the optimal *d_c_* value and obtain the final clustering results to complete the clustering process

### 3.5. Algorithm Time Complexity

For a data set with a sample size of *N*, the time complexity of the DPC algorithm mainly consists of calculating the distance matrix D with a complexity of O(*N2*), sorting the Euclidean distances with a complexity of O(*N2*log2*N*), and computing the local density and relative distance δ with a complexity of O(*N2*). Assuming the maximum range of black widow population is *M*, and the maximum number of iterations is *T*, and the dimension for optimizing the cutoff distance *d_c_* is 1, the complexity of optimizing *d_c_* is O ( M×T ). During the optimization process, as *d_c_* changes, it affects the local density and relative distance δ, resulting in a complexity of O ( N2×T ) for this process. In summary, the time complexity of the algorithm mentioned in this chapter is O(N2(log2*N*+*T*)).

## 4. Experimental Results and Analysis

### 4.1. Experimental Dataset and Experimental Environment

This chapter used 12 datasets, including synthetic and UCI datasets, to validate the proposed clustering algorithm. Table 1 details attributes of the artificial and real datasets, while Table 2 shows the parameter settings for BWDPC, DPC, and K-means algorithms. The experimental environment consists of a LENOVO (Riyadh, Saudi Arabia) desktop computer with Windows 10 64-bit operating system, an Intel i7-10700 processor (Santa Clara, CA, USA), Python 3.9 as the programming environment, PyCharm as the development tool, and 8 GB of RAM.

### 4.2. Experiments on Synthetic Datasets

For the datasets provided in Table 1, BWDPC, DPC, DBSCAN, and K-means were used for clustering. Figure 3, Figure 4 and Figure 5 show the clustering results of the four algorithms on the R15 dataset, Aggregation dataset, and D31 dataset, respectively. These three datasets have different overall distributions and numbers of clusters, which can more intuitively reflect the clustering performance of the four algorithms. Points with different colors in the figures are assigned to different clusters.

The clustering results for the Aggregation dataset in Figure 4 reveal that only the BWDPC algorithm accurately clusters the dataset, while the other three algorithms fall short of achieving precise clustering. Due to an incorrect choice of *d_c_*, the DPC algorithm erroneously divides the blue cluster in the top-left corner into two, and the red cluster in the bottom-left corner, originally two clusters, is treated as one, resulting in substantial errors. While K-means correctly determines the number of clusters, it falls short of achieving accurate clustering. Specifically, K-means generates two cluster centers within one cluster and places cluster centers between the black clusters, whereas points in the red and pink clusters should belong to the same cluster. This incorrect setting of cluster centers by K-means results in substantial errors. Even though DBSCAN accurately identifies the number of clusters, some points are erroneously marked as noise. For instance, certain boundary points of the purple cluster in the top-left corner are incorrectly classified as noise, leading to a slightly inferior clustering result.

Figure 3 reveals that only BWDPC and K-means can accurately cluster the R15 dataset. The DPC algorithm exhibits clear errors in choosing cluster centers, leading to unsatisfactory clustering outcomes. DBSCAN erroneously designates certain boundary points as noise, diminishing clustering accuracy. In the D31 dataset, both BWDPC and K-means successfully achieve accurate clustering, whereas DBSCAN not only mislabels certain boundary points as noise but also errs in determining the number of clusters. For instance, the red cluster, which originally belongs to two different clusters, is incorrectly consolidated into one by DBSCAN. DPC also faces analogous problems due to the absence of a suitable *d_c_* value, leading to substantial errors in cluster centers. In comparison, BWDPC not only determines the correct number of clusters but also identifies the positions of cluster centers more accurately, resulting in superior clustering performance.

Figure 6 and Figure 7 show the clustering results of the four algorithms on the Two_cluster dataset and Five_cluster dataset, respectively. These datasets further demonstrate the accuracy of the BWDPC algorithm in clustering.

From the clustering results in Figure 6 and Figure 7, it can be seen that BWDPC algorithm, DPC algorithm, and K-means algorithm can accurately cluster the datasets and find cluster centers. However, the DBSCAN algorithm treats the boundary points as noise and produces a wrong classification of the data points in the lower left corner, which indicates that the DBSCAN algorithm may produce incorrect classification when dealing with uneven data density, resulting in suboptimal clustering performance.

Table 3 provides clustering evaluation metrics for BWDPC and other comparison algorithms on six datasets. From the evaluation metrics in Table 3, it can be observed that BWDPC, with the improvement in cluster center selection strategy and the optimization of the cutoff distance using the Black Widow Optimization Algorithm, achieved good results on most datasets. Furthermore, all the clustering metrics of the BWDPC algorithm outperformed those of the DPC algorithm, indicating the significant effect of optimizing the cutoff distance *d_c_* in BWDPC. The best results are shown in bold, and the clustering metrics used in this section are *FMI*, *ARI*, and *AMI*.

### 4.3. Experiments on Real-World Datasets

The experiment used six real-world datasets to test the performance of the BWDPC algorithm. These datasets have different sample sizes, feature numbers, and cluster quantities. Table 1 provides specific information for each real dataset. The experiment conducted clustering using BWDPC, DPC, DBSCAN, and K-means on these six datasets, and the results are shown in Table 3, with the best results highlighted in bold.

As shown in Table 4, BWDPC excels over other clustering algorithms in six real datasets and shows substantial improvements on certain UCI datasets. For example, BWDPC performs exceptionally well, securing a leading position in clustering results, especially on widely used Iris and Sym datasets. In comparison to the DPC algorithm, BWDPC improves *FMI*, *ARI*, and *AMI* scores by 0.08, 0.2, and 0.1, respectively, on the Iris dataset. Specifically, the Sym dataset sees improvements of 0.01, 0.02, and 0.01 in *FMI*, *ARI*, and *AMI* scores, respectively. The sensitivity is due to DPC’s responsiveness to the truncation distance parameter (*d_c_*) in small-sample datasets. However, on the Segment dataset, despite BWDPC’s improved clustering performance over DPC, with increases of 0.10, 0.12, and 0.20 in *FMI*, *ARI*, and *AMI* scores, respectively, the overall clustering performance remains unsatisfactory. This is because BWDPC faces challenges when handling high-dimensional data, which may lead to less satisfying results. Besides the mentioned datasets, the BWDPC algorithm achieves optimal clustering results on the Segment and Zoo datasets. Specifically, on the Zoo dataset, BWDPC outperforms the DPC algorithm with increases of 0.31, 0.34, and 0.2 in *FMI*, *ARI*, and *AMI* scores, respectively, showcasing robust performance in low-dimensional data. In comparison to the PDC algorithm, BWDPC automatically optimizes the *d_c_* value, resulting in optimal clustering results. The enhanced BWDPC algorithm can precisely identify true cluster centers. In contrast to the K-means algorithm, both BWDPC and DPC can accurately identify cluster centers in streaming datasets. In certain datasets, DBSCAN might misclassify boundary points as noise, leading to less accurate clustering results.

## 5. Conclusions

In this study, BWDPC utilizes the Black Widow Optimization Algorithm to dynamically determine the optimal cutoff distance *d_c_*, thereby improving clustering performance. Moreover, by introducing a search factor and dynamically updating the spider population range, the algorithm addresses the challenge of parameter specificity, allowing it to avoid local optima and expedite convergence. The results obtained from six artificial datasets and six UCI real datasets illustrate that, based on the comparison of the Fowlkes–Mallows Index (*FMI*), Adjusted Rand Index (*ARI*), and Adjusted Mutual Information (*AMI*), BWDPC consistently and accurately identifies cluster centers, yielding optimal clustering outcomes in most cases. It can be asserted that BWDPC outperforms existing algorithms on the majority of datasets, demonstrating higher accuracy. While the BWDPC algorithm achieves automatic optimization of the cutoff distance *d_c_*, the parameter *K* for the number of cluster centers still requires manual determination. Consequently, additional research is necessary to implement the adaptive selection of the parameter *K*, aiming to decrease empirical input and human involvement while preserving algorithm accuracy and robustness, thus enhancing clustering efficiency. Nonetheless, BWDPC has its limitations. While the algorithm demonstrates proficiency on low-dimensional data, its clustering performance diminishes on high-dimensional data, which also constitutes a key area for future research. BWDPC excels in swiftly identifying irregular-shaped clusters and adeptly adjusting to clusters with varying densities in real-world applications. In summary, by leveraging the strengths of DPC and attaining automatic determination of the *d_c_* parameter, BWDPC has delivered exceptional clustering results.

## Figures and Tables

**Table 1:** Description of artificial synthetic datasets and UCI datasets.

Dataset	Instances	Attributes	Clusters
R15	600	2	15
Aggregation	788	2	7
D31	3100	2	31
Two_cluster	400	2	2
Five_cluster	2000	2	5
Flame	240	2	2
Iris	150	4	3
Wine	178	13	3
Sym	350	2	3
Waveform3	5000	21	3
Segment	2310	18	7
Zoo	266	2	3

**Table 2:** Parameter settings.

Dataset	BWDPC	DPC	DBSCAN	
	*d_c_*	*d_c_*	eps	mpts
R15	0.5533	2.0000	0.3200	3
Aggregation	3.1000	3.2000	1.7600	14
D31	0.2121	2.4000	0.8000	24
Two_cluster	1.6802	1.6000	0.2500	2
Five_cluster	1.2126	1.3000	0.4200	7
Flame	0.3447	4.0000	1.4800	16
Iris	0.2932	3.0000	1.6300	2
Wine	32.3152	3.6000	4.3000	2
Segment	3.6890	1.6000	0.1000	2
Waveform3	3.7420	0.1200	3.6000	2
Zoo	6.9286	1.6000	2.5000	6
Sym	0.0784	0.5000	0.1000	6

**Table 3:** Presents the results for artificial datasets.

Dataset	Metric	BWDPC	DPC	DBSCAN	K-Means
R15	*FMI*	0.9932	0.9220	0.9394	0.9932
	*ARI*	0.9913	9.9144	0.9347	0.9927
	*AMI*	0.9967	0.9672	0.9450	0.9938
Aggregation	*FMI*	0.9982	0.8796	0.9110	0.8159
	*ARI*	0.9978	0.8454	0.8853	0.7624
	*AMI*	0.9956	0.9152	0.8865	0.8776
D31	*FMI*	0.9390	0.6040	0.7901	0.9538
	*ARI*	0.9370	0.5369	0.7826	0.9522
	*AMI*	0.9558	0.8325	0.8882	0.9653
Two_cluster	*FMI*	0.9950	0.9950	0.9651	0.9950
	*ARI*	0.9900	0.9900	0.9315	0.9900
	*AMI*	0.9772	0.9772	0.8833	0.9772
Five_cluster	*FMI*	0.9921	0.9316	0.9387	0.9940
	*ARI*	0.9905	0.9033	0.9137	0.9915
	*AMI*	0.9754	0.8823	0.8480	0.9809
Flame	*FMI*	0.7942	0.6002	0.7511	0.7363
	*ARI*	0.5734	−0.0302	0.5453	0.4534
	*AMI*	0.5672	0.1064	0.5985	0.3969

**Table 4:** Results of the artificial datasets.

Dataset	Metric	BWDPC	DPC	DBSCAN	K-Means
Iris	*FMI*	0.8407	0.7567	0.7714	0.5835
	*ARI*	0.7592	0.5609	0.5681	0.3711
	*AMI*	0.8032	0.7050	0.7316	0.4227
Wine	*FMI*	0.5834	0.5674	0.5354	0.5039
	*ARI*	0.3715	0.3016	−0.0003	0.2536
	*AMI*	0.4131	0.4169	0.0502	0.3600
Segment	*FMI*	0.4618	0.3808	0.3047	0.4370
	*ARI*	0.3204	0.2013	0.0001	0.3133
	*AMI*	0.4927	0.3920	0.0780	0.4518
Waveform3	*FMI*	0.5338	0.5011	0.4152	0.5039
	*ARI*	0.2817	0.1584	0.0029	0.2536
	*AMI*	0.3512	0.2467	0.0587	0.3620
Zoo	*FMI*	0.8136	0.5076	0.4839	0.7741
	*ARI*	0.7197	0.3796	0.0075	0.7087
	*AMI*	0.7598	0.5687	0.0079	0.7527
Sym	*FMI*	0.8333	0.8239	0.4793	0.7369
	*ARI*	0.7357	0.7178	0.2888	0.5335
	*AMI*	0.7727	0.7626	0.4719	0.5645

## Data Availability

Data are contained within the article.
